# New Insights Into Structure and Function of *TIFY* Genes in *Zea mays* and *Solanum lycopersicum*: A Genome-Wide Comprehensive Analysis

**DOI:** 10.3389/fgene.2021.657970

**Published:** 2021-05-12

**Authors:** Parviz Heidari, Sahar Faraji, Mostafa Ahmadizadeh, Sunny Ahmar, Freddy Mora-Poblete

**Affiliations:** ^1^Faculty of Agriculture, Shahrood University of Technology, Shahrood, Iran; ^2^Department of Plant Breeding, Faculty of Crop Sciences, Sari Agricultural Sciences and Natural Resources University (SANRU), Sari, Iran; ^3^Minab Higher Education Center, University of Hormozgan, Bandar Abbas, Iran; ^4^Institute of Biological Sciences, University of Talca, Talca, Chile

**Keywords:** *JAZ* genes, ZIM subfamily, expression profile, *in silico* study, phylogenetic analysis

## Abstract

The *TIFY* gene family, a key plant-specific transcription factor (TF) family, is involved in diverse biological processes including plant defense and growth regulation. Despite TIFY proteins being reported in some plant species, a genome-wide comparative and comprehensive analysis of *TIFY* genes in plant species can reveal more details. In the current study, the members of the *TIFY* gene family were significantly increased by the identification of 18 and six new members using maize and tomato reference genomes, respectively. Thus, a genome-wide comparative analysis of the *TIFY* gene family between 48 tomato (*Solanum lycopersicum*, a dicot plant) genes and 26 maize (*Zea mays*, a monocot plant) genes was performed in terms of sequence structure, phylogenetics, expression, regulatory systems, and protein interaction. The identified TIFYs were clustered into four subfamilies, namely, TIFY-S, JAZ, ZML, and PPD. The PPD subfamily was only detected in tomato. Within the context of the biological process, *TIFY* family genes in both studied plant species are predicted to be involved in various important processes, such as reproduction, metabolic processes, responses to stresses, and cell signaling. The Ka/Ks ratios of the duplicated paralogous gene pairs indicate that all of the duplicated pairs in the *TIFY* gene family of tomato have been influenced by an intense purifying selection, whereas in the maize genome, there are three duplicated blocks containing Ka/Ks > 1, which are implicated in evolution with positive selection. The amino acid residues present in the active site pocket of TIFY proteins partially differ in each subfamily, although the Mg or Ca ions exist heterogeneously in the centers of the active sites of all the predicted TIFY protein models. Based on the expression profiles of *TIFY* genes in both plant species, JAZ subfamily proteins are more associated with the response to abiotic and biotic stresses than other subfamilies. In conclusion, globally scrutinizing and comparing the maize and tomato *TIFY* genes showed that *TIFY* genes play a critical role in cell reproduction, plant growth, and responses to stress conditions, and the conserved regulatory mechanisms may control their expression.

## Introduction

Transcription factors (TFs) are main regulatory proteins in whole living cells that bind to DNA flanking target genes. An interaction occurs among transcriptional regulators, consisting of chromatin-modifying or remodeling proteins, activating or repressing transcription. In plants, TFs play a significant role in regulating gene expression and plant responses to environmental conditions. *TIFY* is a particular gene family that is annotated as TFs by its function, also formerly named ZIM (zinc-finger protein expressed in inflorescence meristem; Nishii et al., 2000; Aparicio-Fabre et al., 2013), and is involved in diverse biological processes including plant defense and growth regulation (Xia et al., 2017; Liu et al., 2020). Members of the *TIFY* gene family contain a common TIFY domain with the conserved motif TIF[F/Y]XG (Vanholme et al., 2007; Xia et al., 2017). *TIFY* genes are divided into four subfamilies: TIFY subfamily, jasmonate ZIM (JAZ), ZIM-like (ZML), and PEAPOD (PPD) (Bai et al., 2011). The members of the TIFY subfamily possess only the TIFY domain, whereas the JAZ subfamily, in addition to the TIFY domain, has a Jas motif (SLX2FX2KRX2RX5PY) near the C-terminus (Staswick, 2008). ZIM (zinc-finger expressed in inflorescence meristem), and ZML proteins to gether belong to the ZML subfamily, which contains a CCT domain (CONSTANS, CO-like, and TOC1) and a C2C2-GATA zinc-finger domain (Saha et al., 2016). Proteins of the PPD subfamily have a PPD domain in the N-terminals and a changed Jas motif, which replaces the conserved proline–tyrosine in their C-terminals (Chung et al., 2009).

The JAZ proteins suppress TFs such as MYC2, which plays a role in promoting jasmonic acid (JA)–responsive gene transcription in plant cells with low levels of JA. The NINJA/TPS (novel interactor for JAZ/TOPLESS) corepressor complex engages as a molecular mechanism in the suppression of the downstream genes (Pauwels et al., 2010). The JAZ protein plays an important role in the JA signaling pathway (Garrido-Bigotes et al., 2019). The level of JA in a plant cell that is growing naturally is low. The level of JA-isoleucine (JA-Ile) expression is usually enhanced once a plant is subjected to harsh conditions or in development. JAZ proteins link to coronatine-insensitive 1 (COI1) via Skp1/Cullin1/F-box protein COI1 (SCFcoi1) complex-mediated ubiquitination and adjust ubiquitin-26S proteasome destruction. JAZ protein synthesis is induced by JA to prevent TFs’ activity (Katsir et al., 2008; Sun et al., 2017; Garrido-Bigotes et al., 2019). The COI1, MYC2, and JAZ performance in JA signaling resembles the main components of the auxin signaling pathway (Katsir et al., 2008). Furthermore, some *TIFY* gene family members are involved in modulating the signaling pathways of hormones such as JA and abscisic acid (ABA) (Zhang et al., 2012; Sirhindi et al., 2016). JAZ directly adjusts plant flower initiation, morphology, tanshinone biosynthesis, salvianolic acids, and cotton fiber initiation (Boter et al., 2015; Hu et al., 2016; Pei et al., 2018; Yu et al., 2018). To date, *TIFY* genes have been recognized in various species, for instance, 27 *TIFY* genes in maize (Bai et al., 2011), 18 in *Arabidopsis* (Chung and Howe, 2009), 49 in wheat (Ebel et al., 2018), and 20 in rice (Chung and Howe, 2009). Some functional studies of the *TIFY* gene family have been performed. *AT4G14720* (PPD2) and *AT4G14713* (PPD1) are engaged in the coordination of leaf growth (White, 2006). *AT4G24470* (ZIM) adjusts hypocotyl and petiole elongation through mediating cell elongation (Shikata et al., 2004), whereas *AT1G51600* (ZML2) plays a transcriptional repressor role in the lignin biosynthesis of transgenic maize (Vélez-Bermúdez et al., 2015).

*TIFY* genes play a remarkable role in leaf growth synchronization and phosphorus-starvation adaptation in *Arabidopsis* and common bean (White, 2006; [Bibr B4][Bibr B79]). In rice, *OsTIFY11b* (*OsJAZ10*) and *OsTIFY3* (*OsJAZ1*) are regulators governing grain size and spikelet development (Hakata et al., 2012; Cai et al., 2014). TIFY proteins are broadly involved in the plant response to abiotic and biotic stresses such as *Pseudomonas syringae DC3000* (*jaz10* mutants) in *Arabidopsis* (Demianski et al., 2012) and bacterial blight resistance (*OsJAZ8*) in rice (Taniguchi et al., 2014). *TaJAZ1* overexpression leads to increased bread wheat resistance in biotic stress (Jing et al., 2019); *GhJAZ2* overexpression leads to increased sensitivity in transgenic cotton under salt stress (Sun et al., 2017). *AtTIFY10a*, *10b*, and *GsTIFY10a* (as their wild soybean homologs) and *OsJAZ8* (in rice) overexpression play significant adjusting roles in the responses of the plant under alkaline and salt stresses, respectively (Zhu et al., 2014; Peethambaran et al., 2018). All of these results indicate that the *TIFY* gene family has multiple regulatory roles in cell signaling and regulating plant responses to stresses and so might be precious resources for stress-responsive genes.

Several studies have examined the function of this gene family’s members, but many structural and regulatory aspects of this gene family remain unknown. Tomato and maize are among the most valuable plants, being important in the human food supply. Previously, 30 *TIFY* genes in maize (Zhang L. et al., 2015) and 20 genes in tomato (Chini et al., 2017) were identified; the studies were mostly accomplished based on gene expression and phylogeny studies. However, in the current study, the number of *TIFY* genes was elevated using the updated reference genomes of maize and tomato, which comprised 18 and six new members of the *TIFY* gene family in maize and tomato, respectively. Hence, a genome-wide comparison analysis of the *TIFY* gene family between tomato (*Solanum lycopersicum*) as a dicot and maize (*Zea mays*) as a monocot was performed based on sequence structure, expression, regulatory systems, and protein interaction. Overall, the reported results increase our knowledge of the evolutionary and regulatory mechanisms of the TIFYs and lay the basis for revealing the mechanism of regulatory and future functional analyses related to molecular mechanisms of *TIFY* genes.

## Materials and Methods

### Identification of *TIFY* Gene Family Members

Reference genomes of *Z. mays* and *S. lycopersicum* were obtained from the Ensembl platform (Bolser et al., 2017) for the detection of TIFY family members. The hidden Markov model search was fulfilled through the TIFY domain (PF06200) in the query box of the HMMER 3.0 program (*E* < × 10^––5^), and the retrieved amino acid sequences were assessed using the SMART^1^ (Schultz et al., 2000) and Pfam^2^ (Finn et al., 2010) databases for the identification of the particular TIFY domain. Genomic sequences and the corresponding cDNA of the predicted proteins were also determined using the Phytozome v13.1 database^3^ (Goodstein et al., 2012), and the locations of *TIFY* genes on chromosomes were determined using the gene ID in Ensembl Plants (Bolser et al., 2017). The ProtParam program on the ExPASy server^4^ (Gasteiger et al., 2003) was used to specify the physicochemical characteristics of the TIFY family proteins, such as theoretical isoelectric point (pI) and molecular weight (MW). The CELLO2GO (Yu et al., 2014) and Gene Ontology (GO) (Yu et al., 2006) programs were used at an *E* < 0.001 to determine GO annotation of *TIFY* genes and for proteins subcellular localization.

### Chromosomal Mapping, Gene Duplications, and Estimation of Ka/Ks Ratio in the Duplicated Pairs

The retrieved *TIFY* genes were mapped onto the maize and tomato chromosomes according to their predicted positions through Ensembl using MapChart software (Voorrips, 2002). The duplication events among the genes were identified using the alignment of the TIFY coding DNA sequences via ClustalW^5^ (Larkin et al., 2007). The matrix with the aligned CDS sequences was predicted by BioEdit software (v. 7.2.5) (Hall, 1999). Genes were considered to be duplicated when there was more than 85% identity at their nucleotide sequences (Zheng et al., 2010), which were manually marked on the chromosomal location. The sequence duplications among species were then determined via the Plant Genome Duplication Database using the MCScan v0.8 program (Wang et al., 2012).

The pressure of selection on the duplicated pairs and dividing of homologous *TIFY* genes were computed by calculating the synonymous (Ks) and nonsynonymous (Ka) exchange rate per site among the gene pairs using the DnaSP v6 software (Rozas et al., 2017). The time of dividing and duplication was appraised by a synonymous mutation rate of λ substitutions per synonymous site per year as *T* = (Ks/2λ (λ = 6.5 × 10^–9^)) × 10^–6^ (Yang et al., 2008). The syntenic relationships of *TIFY* genes among the orthologous pairs of maize–rice and tomato–*Arabidopsis* at both gene and chromosome levels were visualized using the Circos software (Krzywinski et al., 2009).

### Phylogenetic Analysis, Motif Recognition, and Promoter *cis*-Elements

Phylogenetic trees of the evolutionary relationships between TIFY protein sequences from maize, rice, barley (*Hordeum vulgare*), tomato, soybean (*Glycine max*), and *Arabidopsis* (downloaded from the Ensembl platform) were constructed using the ClustalW method and maximum likelihood (ML) algorithm with 1,000 bootstrap replications, implemented in the MEGA 6.0 software (Tamura et al., 2013). Finally, the phylogenetic tree was drawn using an interactive tree of life (Letunic and Bork, 2019). The MEME software^6^ (Bailey et al., 2009) was used to identify the conserved motifs in amino acid sequences of TIFYs based on the following setting: motifs number: 15; minimum width: 6; maximum width: 50.

### Promoter Analysis and Protein–Protein Interaction Assay

*cis*-Elements within the promoter region of *TIFY* genes were identified using the 2,000-bp upstream region of the ATG start codon in each putative *TIFY* gene on the PlantCARE server http://bioinformatics.psb.ugent.be/webtools/plantcare/html/^7^ (Lescot et al., 2002). The STRING v11 program http://stringdb.org^8^ (Szklarczyk et al., 2019) was used to determine key *TIFY* genes in the studied plant species, considering GO annotations, and to infer protein-protein interaction networks.

### Three-Dimensional Protein Modeling and Molecular Docking of the Protein Pocket Sites

The three-dimensional (3D) protein structures associated with some candidate TIFY proteins of the identified subfamilies were built via iterative template-based fragment assembly simulations in I-TASSER (Yang et al., 2015). The best models were purified using the 3D-refine program (Bhattacharya et al., 2016). Then, the predicted structures were validated using a Ramachandran plot via measuring the backbone dihedral phi (ϕ) and psi (Ψ) angles using the RAMPAGE program (Lovell et al., 2003). For the prediction of the protein pockets and cavities, the refined structures of TIFY proteins were analyzed using P2Rank in the PrankWeb software (Jendele et al., 2019) and the CASTp tool (Tian et al., 2018). Finally, the results were visualized in PyMOL (DeLano, 2002).

### *In silico* Expression Analysis of TIFY Genes Through RNA-Seq Data

For the assessment of maize and tomato *TIFY* gene transcription, available RNA-seq data were used to retrieve data in response to stimuli in multiple tissues. The fragments per kilobase of exon per million fragments mapped (FPKM) expression values in maize various tissues, in addition to under stimuli exposure, were determined in the maize B73 v4 genome through a gene ID (Zm00001) search in qTeller in MaizeGDB (Portwood et al., 2019) using the previously published reports for multiple tissues (accession IDs PRJNA171684 and SRP010680) (Stelpflug et al., 2016) and under stresses with accession numbers GSE71046 (Forestan et al., 2016) and PRJNA244661 (Waters et al., 2017). For the expression values of *TIFY* genes in tomato, the RNA-seq transcriptome data related to various tissues in the tomato cultivar Heinz and tomato leaves treated with different bacteria and pathogen-associated molecular patterns were extracted from the tomato functional genomics database^9^ (Fei et al., 2010). The extracted magnitudes were then log2 transformed and used to generate the heatmaps and Venn diagrams via the TBtools package (Chen et al., 2020). Clustering of heatmaps was performed using complete data and the Euclidean distance method.

## Results

### Identification of *TIFY* Genes and GO Annotation

**FIGURE 1:**
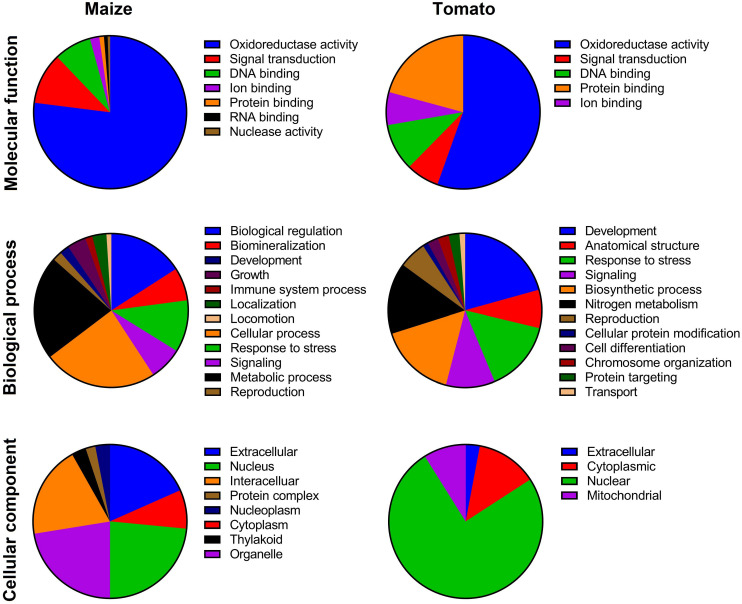
Gene Ontology (GO) of TIFY family members based on molecular function, biological process, and cellular component in maize and tomato. The GO terms were assigned based on a protein sequences search in the CELLO2GO program.

Based on the HMMER result, 48 and 26 nonredundant putative TIFY proteins were identified in the *Z. mays* and *S. lycopersicum* genomes, respectively (Supplementary Table 1). According to the protein-specific domain, the recognized TIFY proteins were classified into four subfamilies: JAZ, TIFY-S (also named TIFY), PPD, and ZML; however, the PPD subfamily was not identified in the maize genome (Supplementary Table 1). The putative TIFY proteins in maize ranged from 60 (in Zm00001d024455) to 539 (in Zm00001d005726) amino acids in length, with the MWs ranging from 6.65 to 57.69 kDa in these proteins, respectively. The theoretical pI of the maize TIFY proteins ranged from 4.37 (in Zm00001d048264) to 10.88 (in Zm00001d032009). The identified TIFY proteins in tomato also ranged from 61 (Solyc01g011175) to 427 (Solyc06g065650), with the MW ranging from 6.95 to 44.85 kDa in these proteins, respectively, and the pI values varied from 4.99 (Solyc01g009730) to 10.34 (Solyc01g097060). Most of the identified TIFY proteins in both of the candidate species revealed an alkaline nature (∼73% in maize and ∼69% in tomato; Supplementary Table 1). The results of subcellular localization revealed that the majority of maize TIFY proteins are localized in the nucleus, extracellularly, intracellularly, and organelles (Figure 1), whereas most tomato proteins showed potential to be located in the nucleus, cytoplasm, and mitochondria (Figure 1).

The evaluation of the biological processes mediated by TIFYs evidenced that most of the proteins are probably implicated in growth and developmental processes and response to stimuli in both monocot and dicot plant species (Figure 1). Among the TIFY family proteins, ∼2 and 18% of TIFY proteins showed potential involvement in the regulation of development in maize and tomato, respectively (Figure 1). The roles of TIFYs in reproduction (∼2% in maize and 5% in tomato) and cellular component metabolism (11% in maize and 21% in tomato) were determined through the GO assay. Most of the recognized TIFYs in both plant species were predicted to be involved in the response to adverse conditions. For instance, 8 and 11% of TIFYs in maize and 10 and 14% of these genes in tomato were assumed to be modulating genes during signaling and stress responses, respectively. In the context of molecular functions, most of the TIFY proteins showed potential involvement in oxidoreductase activity (79% in maize and 56% in tomato) and DNA-binding activity (8% in maize and 10% in tomato) (Figure 1). The potential involvement of some TIFY proteins in ion binding (2% in maize and 6% in tomato) was also expected, in addition to their molecular functions in the cell.

### Chromosomal Distribution and Gene Duplications

**FIGURE 2:**
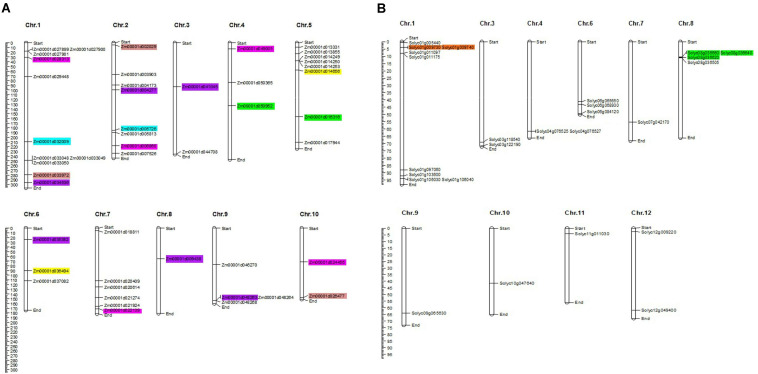
Chromosomal distribution of *TIFY* genes predicted from the *Zea mays* **(A)**, and *Solanum lycopersicum* **(B)** genomes. The graphical genetic maps were created via the MapChart software, and the duplicated gene pairs are highlighted in the same color.

Uneven distribution of the *TIFY* genes was predicted on 10 maize chromosomes with 11, eight, and eight genes on chromosomes 1, 2, and, 5, respectively (Figure 2A). Chromosomes 3, 8, and 10 in maize also accounted for only two, one, and two *TIFY* genes, respectively. In the tomato genome, *TIFY* genes were distributed on 10 of 12 chromosomes, with a high density on chromosomes 1 and 8, which contained nine and four genes, respectively (Figure 2B). Sixteen and three duplicated gene pairs were identified in the TIFY family in maize and tomato, which clustered into five and two groups, respectively (Supplementary Table 2). The segmental duplication events were found to be higher in the maize genome than tomato’s. Among the duplicated clades in maize, groups A, C, and D showed significant duplication events with five, four, and four gene pairs, respectively (Figure 2A and Supplementary Table 2). The duplicated gene pairs in tomato were localized only at chromosomes 1 and 8 (Figure 2B and Supplementary Table 2), whereas the duplicated pairs in maize were found to be distributed on all chromosomes with the highest range of duplication on chromosomes 1 and 2. The intraspecies synteny analysis revealed that all of the duplicated blocks in tomato are collinear, such as *Solyc01g009730* and *Solyc01g009740*. The Ka/Ks ratios of the duplicated paralogous gene pairs cover a domain from 0.491 to 1.830 in maize and 0.570 to 0.775 in tomato, whereas in the maize genome, there are three duplicated blocks containing Ka/Ks > 1 (Supplementary Table 2).

### Phylogenetic Relationships and Conserved Motifs

**FIGURE 3:**
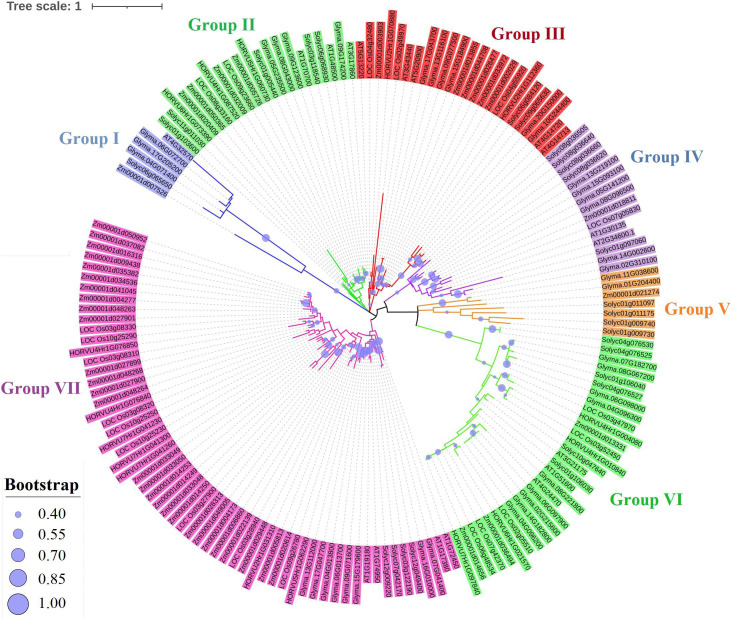
Phylogenetic analysis of TIFY proteins from monocots (maize, barley, and rice) and dicots (tomato, *Arabidopsis*, and soybean) based on the maximum likelihood (ML) method.

**FIGURE 4:**
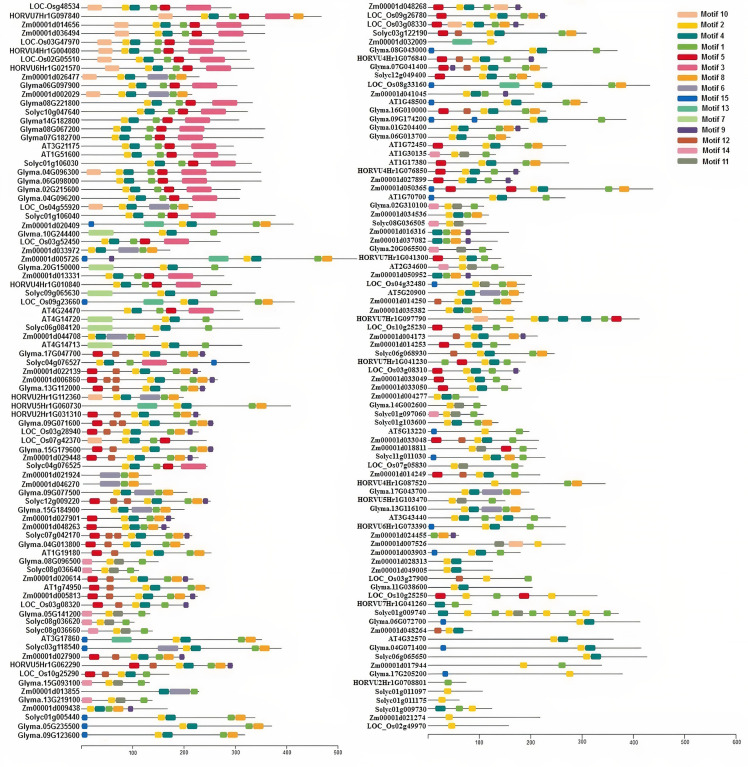
Conserved motif distribution in TIFY proteins from monocots (maize, barley, and rice) and dicots (tomato, *Arabidopsis*, and soybean). A total of 15 conserved protein motifs were predicted via the MEME program with a length of 6 to 50 amino acid residues. The proteins in the same clade have a similar motif pattern in their structure.

A total of 169 TIFY protein sequences from three monocot plant species, namely, maize, rice, and barley, along with three dicot species, including tomato, *Arabidopsis*, and soybean, were employed to assay the phylogenetic relationships that clustered all TIFYs into the seven different groups (Figure 3). Based on the conserved protein motifs, three of 15 motifs (motifs 1, 2, and 3) represent the specific TIFY protein domains in all candidate species (Figure 4). The presence of only motif 2 demonstrates the important functional part of the TIFY-S class; motif 2 with motif 1 represents the JAZ clade, which includes more proteins; and the presence of motifs 1, 2, and 3 demonstrates the ZML group of the TIFY family (Figures 3, 4). In addition, some conserved motifs were observed on the outside of the protein domain. The TIFY proteins belonging to the same phylogenetic class also have some conserved motifs beyond the specific domain region. For instance, motif 7 is shared by the members of the JAZ subfamily. Hence, the motif architectures are approximately conserved in each TIFY-S subfamily, which refer to the conserved and specific functions of the proteins in these clusters. Overall, the members of the JAZ subfamily show high diversity, suggesting relative evolutionary conservation in the cellular function of these proteins from various plant species. High diversity between *TIFY* genes was observed, indicating that the *TIFY* gene family originated before the divergence of monocots and dicots.

### Identification of Blocks Duplicated Between Species With Estimation of the Ka/Ks Ratios

**FIGURE 5:**
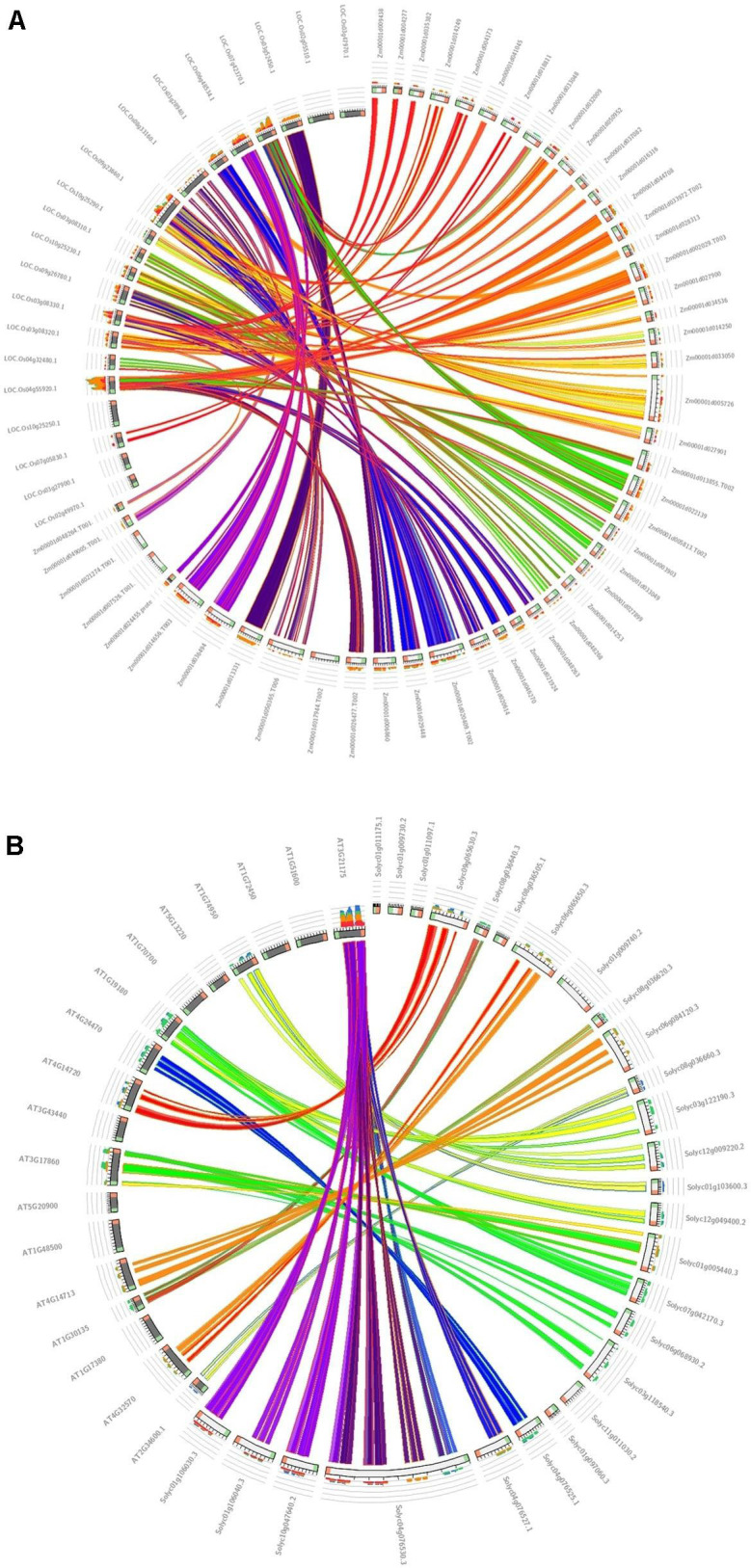
Synteny analysis of *TIFY* genes. The syntenic blocks of maize *TIFY* genes are compared with the monocot model plant (*O. sativa*) **(A)**, and the syntenic blocks of tomato are compared with the dicot model plant (*A. thaliana*) **(B)**.

The association of positive Darwinian selection in duplication and divergence, an important parameter for studying the effects of positive selection engagement in gene divergence (Yang et al., 2008), was calculated for the duplicated *TIFY* genes in maize in comparison with the monocot model plant (*Oryza sativa*) (Figure 5A) and tomato compared with the dicot model plant (*Arabidopsis thaliana*) (Figure 5B) as their closest orthologous genes (Supplementary Table 3). As a result, 11 duplicated and 10 triplicated blocks in maize compared with rice species, and three duplications and one triplication in tomato in comparison with *Arabidopsis*, were identified; the average Ka/Ks ratio for the diverged blocks was estimated to be 0.450 and 0.427 (<1) in maize and tomato, respectively. The duplication event for the *TIFY* genes was estimated to have occurred approximately 32–137 MYA between maize and rice and 109 to 150 MYA between tomato and *Arabidopsis* (Supplementary Table 3). Among the closest orthologous *TIFY* in the grass species, the relatively higher rate of synonymous substitution between maize and rice suggests their earlier divergence about 63 MYA compared to that between tomato and *Arabidopsis* genes (around 124 MYA). Therefore, the duplication and divergence events among the *TIFY* genes from monocot species can be considered as a significant aspect in the evolution of this gene family.

### Promoter *cis*-Elements and Protein–Protein Interaction Network

**FIGURE 6:**
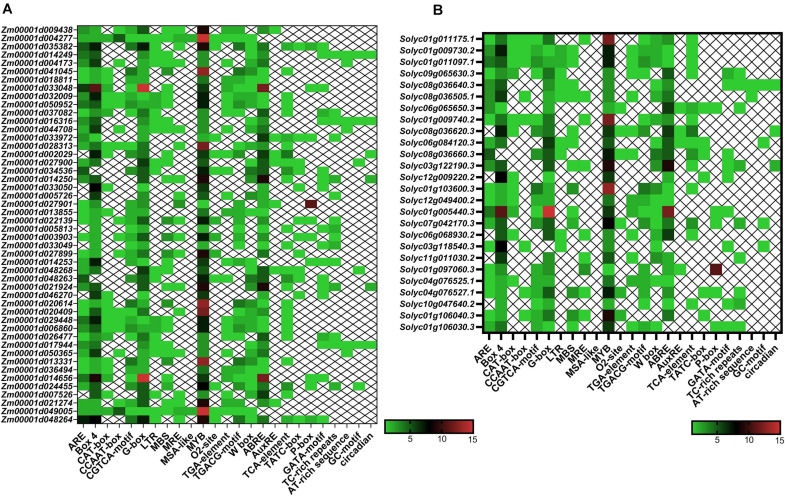
Heatmaps of *cis*-regulatory elements distribution in the promoter region of *TIFY* genes of maize **(A)** and tomato **(B)**. The upstream region (2,000 bp) of the ATG start codon in each putative *TIFY* gene promoter region was analyzed using the PlantCARE server (Lescot et al., 2002).

In the present study, several kinds of *cis*-elements that deal with various phytohormones, abiotic stimulus conditions, and regulation of development were identified in the promoter of *TIFY* genes (Figure 6). The ABA responsiveness (ABRE), ethylene responsiveness (ERE), and methyl jasmonate responsiveness (MeJA) factors were observed as highly occurring hormone-responding *cis*-elements approximately in the *TIFY* genes promoter. The light-responsive G-Box and Box 4, wounding stress-responsive WUN-motif, and stress-responsive MYB elements were detected as the other regulatory *cis*-elements frequently distinguished in the *TIFY* genes promoter area, suggesting the important roles of this gene family in stress management in monocot and dicot crops. Moreover, observation of the MBS element, the MYB protein binding region engaged in drought stress coping, and regulation of the flavonoid biosynthetic genes, in some *TIFY* genes, such as Zm00001d041045, Zm00001d022139, Zm00001d003903, and Zm00001d013331 in maize and Solyc01g103600, Solyc07g042170, Solyc04g076527, and Solyc01g106040 in tomato, confirmed the important role of these genes in anthocyanin/flavonoid production and stimulus coping. The TC-rich repeats (regulating defensive reactions), low-temperature responsive element, TCA element (salicylic acid–responsive), TGA element (auxin-responsive), and W-Box (WRKY TF-binding region, important for responses to abiotic stimuli) were detected as the important abiotic/hormone stress–responsive elements significantly detected in most *TIFY* genes. Another important finding was the discovery of multiple regulatory *cis*-elements related to phytohormones and environmental stimuli in the majority of *TIFY* genes, revealing the role of these genes in plant growth and dealing with stress conditions. In general, the results showed that the distribution of regulatory elements in the promoter region of *TIFY* genes is similar in both studied species, and the observation of different regulatory elements indicated that this gene family is involved in different cellular pathways.

**FIGURE 7:**
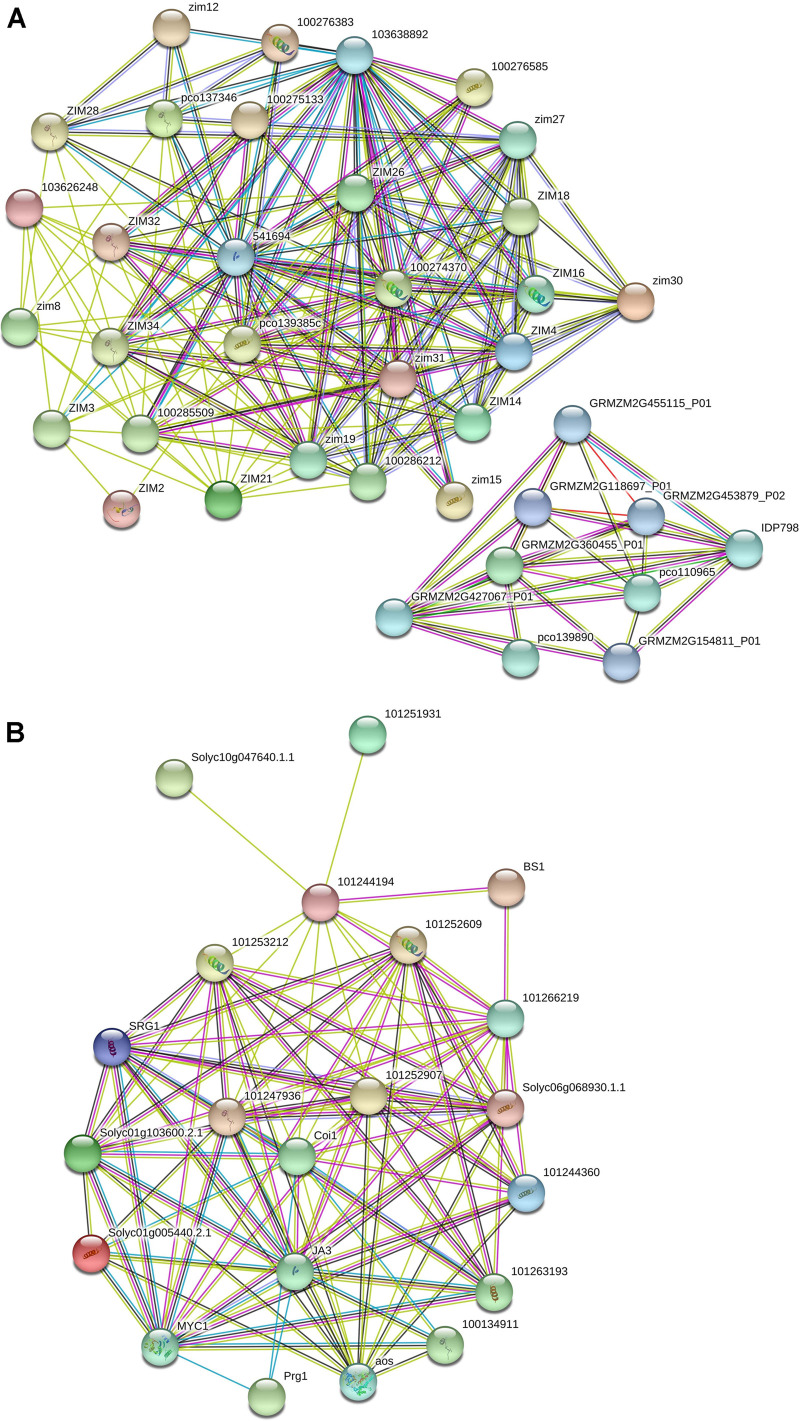
Interaction network of *TIFY* genes in maize **(A)** and tomato **(B)** predicted using the String database.

The interactome data related to *TIFY* genes in maize identified two subnetworks, which showed that TIFY proteins interact with the genes engaged in protein dimerization activity, RNA binding, hydrolase activity, and damaged DNA binding (Figure 7A and Supplementary Table 4). The GRMZM2G455115 and GRMZM2G118697 proteins, which are cleavage and polyadenylation specificity factors, were reported to interact with TIFY proteins to regulate posttranscriptional gene silencing by RNA. The DNA ligases GRMZM2G427067 and GRMZM2G137968, which are single-stranded helicases, also revealed a highly confident interaction with TIFYs in maize that contributes to DNA ligation involved in DNA repair, DNA replication, and cellular response to DNA damage. Furthermore, the protein–protein interaction network in tomato showed the interactions between TIFY proteins and TF MYCs and salt-responsive proteins (Figure 7B and Supplementary Table 5). The BIG SEEDS protein BS1, salt-responsive protein SRG, TF MYC, and Pto-responsive gene *Prg1* showed a significant contribution with *TIFY* genes in the regulation of the defense response, plant hormone signal transduction, and multicellular organism development through a hormone-mediated pathway. The interaction of TIFY proteins with the critical coronatine-insensitive Coi1 and allene oxide synthase regulates ubiquitin-dependent protein catabolic and fatty acid biosynthetic processes, which are essential for protein function and plant reproduction and viability. Therefore, our results reveal that TIFY proteins significantly collaborate with the proteins from various metabolisms, which can regulate plant responses to external stimuli and growth.

### Homology Modeling of TIFY Proteins and Docking Assay of the Pocket Sites

**FIGURE 8:**
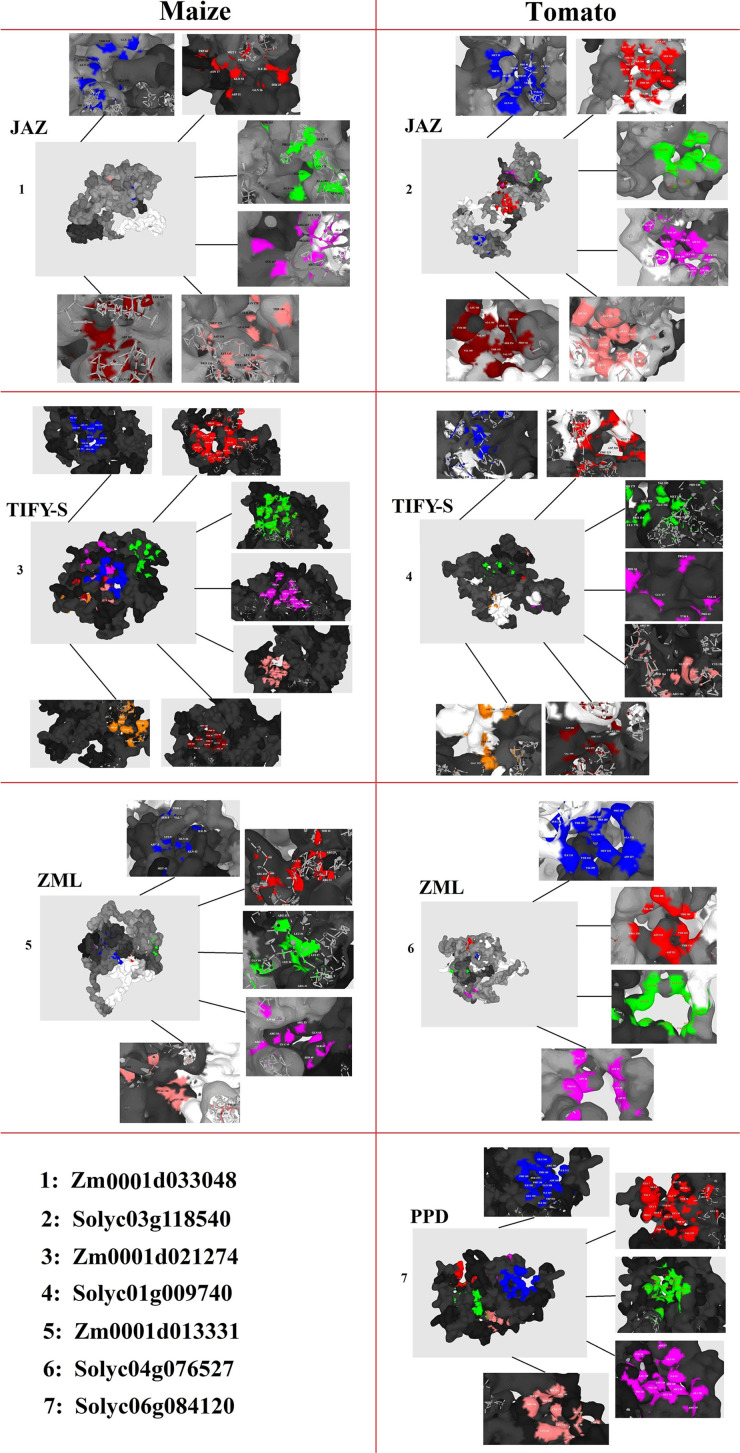
Docking analysis of pocket sites of each subfamily of TIFY proteins including JAZ, TIFY-S, ZML, and PPD.

The 3D structures revealed the presence of the conserved TIFY domain in all of the studied TIFY proteins, which showed a typical 3D frame comprising antiparallel β-sheets followed by parallel α-helixes (Supplementary Figure 1). Topographic features of TIFY proteins were evaluated through the P2Rank program, and major pockets are shown in multiple-colored regions in Figure 8. As a result, different pockets were predicted as the binding region/active sites in the candidate proteins from JAZ, TIFY-S, ZML, and PPD clusters. The amino acid residues present in the pocket sites of TIFY proteins partially differ in each subfamily, although Mg or Ca ions were heterogeneously observed in the center of the active sites of all of the predicted TIFY protein models. In the JAZ subfamily of maize, PRO, ALA, ASN, HIS, ARG, GLY, ASP, and THR were predicted as the important binding residues, whereas SER, THR, PRO, GLU, VAL, LYS, LEU, and TYR were identified as the key residues. Investigation of the predicted pocket sites of TIFY proteins also showed that LEU, CYS, and SER in the TIFY-S of maize and VAL, LYS, GLU, ARG, THR, SER, and LEU in the TIFY-S of tomato have high potential as active binding sites. ARG, THR, LEU, GLN, LYE, and VAL in ZML of maize and SER in ZML of tomato were predicted as the important binding residues. In the PPD subfamily, ARG and GLU showed high potential as key binding residues (Figure 8). Based on our results, the important amino acids found in the pocket sites of all of the candidate TIFY proteins demonstrate the importance of these residues in the positioning on the DNA molecule and, finally, the cellular function performance during various developmental and defensive processes.

### Expression Assay of TIFYs in Multiple Tissues and Under Abiotic/Biotic Stimuli via RNA-Seq Data

**FIGURE 9:**
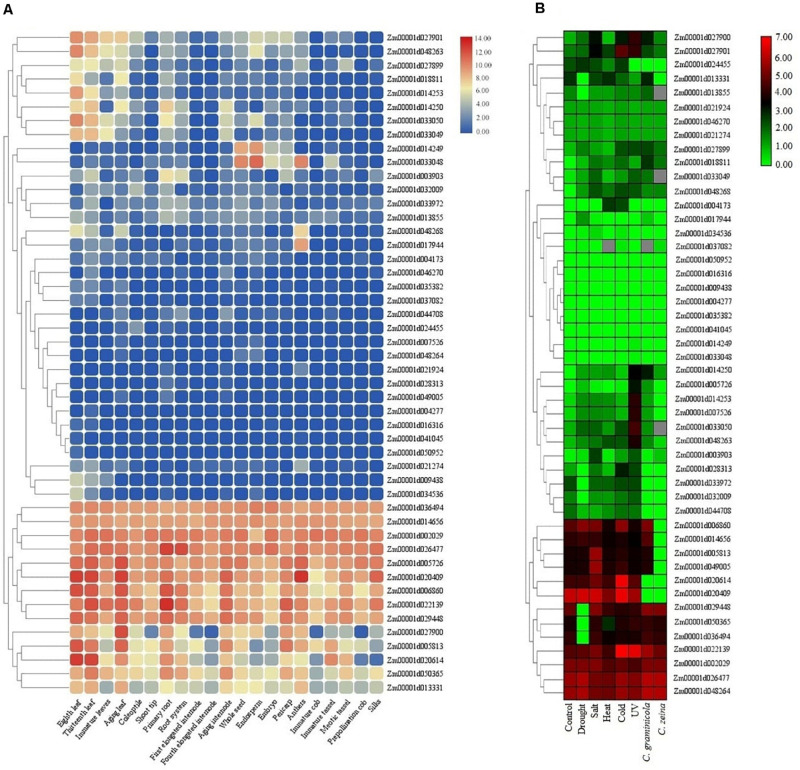
Expression heatmaps of *TIFY* genes of maize in different tissues **(A)** and in response to abiotic and biotic stresses **(B)**. The heatmaps were generated based on the log-2–transformed RNA-seq fragments per kilobase of exon per million fragments mapped (FPKM) magnitudes in the maize genome B73 v4.

The expression patterns of *TIFY* genes were investigated under normal growth in multiple tissues in addition to during stress conditions using RNA-seq data sets in maize and tomato. The FPKM values from various parts of roots, leaves, internodes, and seeds in maize were used for identifying genes differentially expressed in these tissues. The results showed a tissue-specific expression pattern of six, one, one, and one *TIFY* genes in leaf, root, internode, and seed tissues, respectively (Figure 9A). The transcription level of *TIFYs* could be divided into some major expression groups that contained genes preferentially expressed in all or one of the tissues. In addition to nine genes that were not expressed across the tissues (such as *Zm00001d041045*, *Zm00001d016316*, and *Zm00001d004277*), 11 *TIFY* genes (e.g., *Zm00001d036494*, *Zm00001d002029*, and *Zm00001d020409*) displayed significant transcription rates in all of the maize tissues, suggesting control of a broad set of genes at the transcriptional level. According to the RNA-seq data related to stress conditions in maize, *Zm00001d022139*, *Zm00001d002029*, *Zm00001d026477*, and *Zm00001d048264* were recognized as the *TIFYs* expressing under all stimulus situations, revealing their important potential in the stress resistance of maize plants (Figure 9A). A total of eight *TIFYs*, for example, *Zm00001d050952*, *Zm00001d035382*, and *Zm00001d033048*, were not expressed under stress. There were two, one, and one *TIFY* genes specifically expressed under UV, fungal, and cold stresses, respectively. Nine *TIFY* genes were also expressed under salt, drought, heat, and cold stresses. Regarding the results, most of the genes with a tendency to express in response to stimuli were from the JAZ and ZML subfamilies, which may reveal the important roles of these genes in dealing with these stimuli.

**FIGURE 10:**
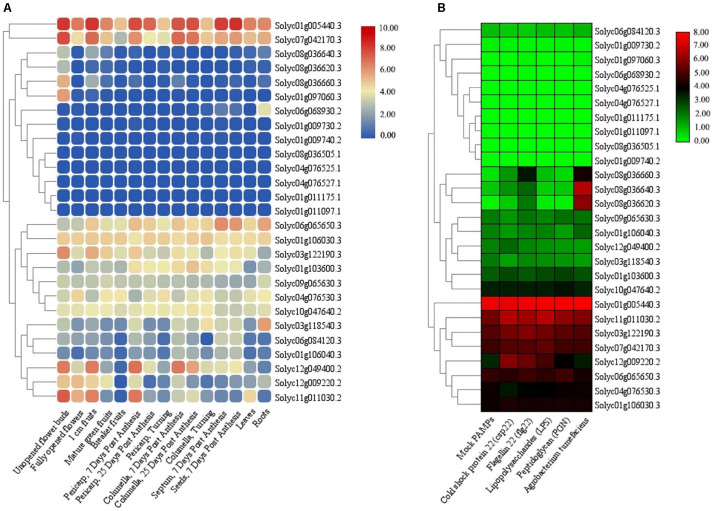
Expression heatmaps of *TIFY* genes of tomato in different tissues **(A)** and in response to abiotic and biotic stresses **(B)**. The heatmaps were generated based on the log-2–transformed RNA-seq FPKM magnitudes in the tomato genome.

The RNA-seq experiments were also employed to further verify the expression of the identified *TIFY* genes in various tissues and under stress in tomato. There were four and one tissue-specific *TIFY* genes in tomato flower and root tissues, respectively (Figure 10A). A total of 11 *TIFY* genes, such as *Solyc01g005440*, *Solyc07g042170*, and *Solyc01g106030*, were significantly expressed in all of the candidate tissues in tomato, whereas seven genes, such as *Solyc01g011175*, *Solyc04g076527*, and *Solyc01g009730*, did not reveal any remarkable transcription level in the tomato tissues. The RNA-seq data under stimulus conditions in tomato revealed one *TIFY* gene responsible for resistance against *Agrobacterium tumefaciens*; the genes *Solyc08g036640* and *Solyc08g036620* also demonstrated remarkable expression under exposure to this bacterium (Figure 10B). Nine *TIFY* genes of tomato were not induced by stress circumstances, whereas 14 genes, such as *Solyc01g005440*, *Solyc11g011030*, and *Solyc06g065650*, demonstrated significant expression in response to all the stimuli (Figure 10B). *Solyc08g036660* from the JAZ clade was found to be significantly up-regulated in coping with *A. tumefaciens* and flagellin 22, suggesting the potential of this gene in dealing with stress. Stress coping in the JAZ protein-encoding genes was significantly greater in comparison with the other subfamilies.

## Discussion

TIFY family proteins, a key TF plant family, have been characterized in different plant species (Vanholme et al., 2007; Aparicio-Fabre et al., 2013; Wang et al., 2020). For instance, 19 members of the *TIFY* gene family in common bean (Aparicio-Fabre et al., 2013), 49 genes in *Triticum aestivum* (Ebel et al., 2018), 36 genes in *Brassica oleracea* (Liu et al., 2020), 36 members in *Brassica rapa* (Saha et al., 2016), 25 genes in poplar (Wang et al., 2020), 20 genes in rice (Ye et al., 2009), *Brachypodium distachyon* (Zhang Z. et al., 2015), 50 members in *Gossypium hirsutum* (Zhao et al., 2016), 34 genes in *Glycine soja* (Zhu et al., 2013), 30 genes in apple (Li et al., 2015), 21 genes in pear (Ma et al., 2018), and 15 genes in watermelon (Yang et al., 2019) have been identified. Furthermore, in previous studies, 30 *TIFY* genes in maize (Zhang L. et al., 2015) and 20 genes in tomato (Chini et al., 2017) were recognized. In the current study, 48 and 26 nonredundant putative *TIFY* genes were identified in the genome of maize (*Z. mays*) and tomato (*S. lycopersicum)*, respectively. Genome size and ploidy level may affect the number of members of a gene family. For instance, bread wheat, a hexaploid species, and *G. hirsutum*, a tetraploid plant, contain the most *TIFY* genes. The identified TIFY proteins are diverse based on their physiochemical properties and domain distribution in maize and tomato. Subcellular localization analysis revealed that the TIFY members of tomato are more located in the nucleus than the TIFY members of maize. Several TIFY members were predicted to be located in organelles, such as chloroplasts and mitochondria (Figure 1). These differences in cellular components indicate that the *TIFY* gene family developed in an extensive regulatory system in plant cells to control various processes (Bai et al., 2011; Cai et al., 2020). Previous studies indicated that the members of the *TIFY* gene family are involved in various mechanisms of plant responses to stress and the regulation of plant growth and development (Hakata et al., 2012; Zhou et al., 2015; Sun et al., 2017; Liu et al., 2020; Wang et al., 2020). In the context of biological processes, the *TIFY* family genes in both candidate plant species are involved in various important processes, such as reproduction, metabolic processes, responses to stresses, and cell signaling (Figure 1). In the context of molecular functions, most identified TIFY family proteins have oxidoreductase activity and DNA-binding TF activity.

High diversity between *TIFY* genes was observed, indicating that the TIFY family genes originated before the divergence of monocots and dicots. Interestingly, the PPD subfamily was only detected in dicots, supporting the hypothesis that *PPD* genes are absent in monocots (Ye et al., 2009; Bai et al., 2011). In *Arabidopsis*, PPD proteins are involved in the regulation of the cell cycle and cell growth (White, 2006). Some other genes in monocots probably compensate for the molecular functions of *PPD* genes (Bai et al., 2011). In the current study, some conserved motifs were detected from outside of the DNA-binding domain regions that may affect the functioning and cellular localization (Heidari et al., 2020; Rezaee et al., 2020). The duplicated gene pair showed different expression levels in response to stress, indicating that duplicated *TIFY* genes probably undergo substantial changes in their regulatory mechanisms and/or sequences to assume novel functions (Faraji et al., 2020).

Gene duplication, an evolutionary event for different species, has a significant role in the enlargement of plant TF families (Freeling, 2009; Wang et al., 2013). In the current study, the Ka/Ks ratios of the duplicated paralogous gene pairs showed that all of the duplicated pairs in the *TIFY* gene family of tomato have been influenced by an intense purifying selection, which could have led to conserved functions or pseudogenization (Juretic et al., 2005), whereas in the maize genome, there were three duplicated blocks containing Ka/Ks > 1, indicating accelerated evolution with positive selection (Faraji et al., 2020). According to intraspecies synteny analysis, all of the duplicated blocks in tomato were collinear, suggesting that these duplication events may have been derived because of the chromosome segmental or large-scale duplication/triplication events (Wang et al., 2018).

Different factors, including interior cavities and protein surface pockets, can affect enzyme activity and DNA–protein interactions (Stank et al., 2016). Prediction of the potential binding sites of proteins can be useful in determining the interaction of proteins and how they are activated (Ahmadizadeh et al., 2020; Faraji et al., 2020). The amino acid residues present in the pocket sites of TIFY proteins partially differ in each subfamily, although the Mg or Ca ions heterogeneously exist in the center of the active sites of all of the predicted TIFY protein models. The SER, GLY, HIS, PRO, GLU, TYR, and ARG amino acids were identified as the important binding residues in the predicted pocket sites of all types of TIFY proteins (Figure 8), which illustrates the potential roles of these proteins in coping with stimuli, in addition to growth and development adjustment, in plant species (Faraji et al., 2020). Proteins with high contents of GLY and PRO residuals play important roles in plants in response to abiotic and biotic stresses (Mousavi and Hotta, 2005). The SER and THR amino acids were predicted as key binding sites in the JAZ protein of tomato. SER, LEU, VAL, and PRO play significantly roles in adjusting the various functions in response to stress (Galili and Höfgen, 2002; Beauregard and Hefford, 2006). The presence of the CYS, VAL, and LYS residues as activating binding sites in almost all TIFY proteins revealed that these proteins may also be involved in sulfur metabolism (Yang et al., 2020). Our results revealed the key binding sites in the protein sequences of each subfamily of TIFY family proteins, which can be used to evaluate the exact function of these proteins. Our findings also indicated that the protein surface pockets in the TIFY family proteins are different in the studied monocot and dicot species and that these differences can affect their associated molecular pathways.

Adverse conditions, such as biotic and abiotic stresses, as limiting factors, affect plant performance. Previous studies revealed that members of the TIFY family, as specific plant TFs, play critical roles in regulating plant responses to adverse environmental conditions (Ebel et al., 2018; Chao et al., 2019; Cai et al., 2020). For instance, overexpression of apple *JAZ2* could significantly improve the tolerance to *P. syringae* pv. *tomato DC3000* in *Arabidopsis* (An et al., 2017). Ebel et al. (2018) found that *TIFY* genes in durum wheat are involved in the response to different stresses, and TIFY proteins may increase germination under salinity treatment. Regarding the results, most of the genes with a tendency to express in response to adverse conditions were from the JAZ subfamily, which may reveal the important roles of this subfamily in dealing with stimuli (Figures 9, 10). Cai et al. (2020) recently stated that the *JAZ* genes of tuber mustard are induced by pathogen *Plasmodiophora brassicae* and salt stress. *JAZ9* in rice can interact with the bHLH062 TF to control salt tolerance via affecting the ion transporter genes (Wu et al., 2015). Similarly, in wheat, five *JAZ* genes were induced under salt stress (Wang et al., 2017). In *G. hirsutum*, 14 *JAZ* genes were induced in response to salinity treatment (Sun et al., 2017). The overexpression of maize *JAZ14* in *Arabidopsis* could increase seedling tolerance to hormone treatments, with ABA and JA, and polyethylene glycol stress (Zhou et al., 2015). Based on the expression profile of *TIFY* genes in both candidate plant species, maize and tomato, we think that *JAZ* subfamily genes are induced to a greater extent in the response to adverse conditions than other subfamilies. It appears that most genes belonging to this common family in both candidate species have similar expression patterns, indicating that the conserved regulatory mechanisms may control their expression.

Various stimuli responses are controlled by the genes’ transcriptional adjustment, which can be modulated by *cis*-elements present in the promoter area (Ahmadizadeh and Heidari, 2014; Heidari et al., 2019). In the present study, several kinds of *cis*-regulatory elements related to cell signaling, the response to biotic and abiotic stresses, and hormone signaling were distinguished in the promoter region of *TIFY* genes. The presence of light-responsive elements, especially G-Box, indicates that light signals can significantly adjust the *TIFY* genes’ transcription, which eventually regulates the genes engaged in defensive lines such as flavonoid biosynthesis pathways (Biłas et al., 2016). The Box 4, ABRE, and MYB elements were frequently distinguished in the *TIFY* genes’ promoter area (Figure 6), suggesting the important roles of this gene family in dealing with stress in monocot and dicot crops. Protein–protein interactions can significantly modulate various cellular functions, such as the replication and transcriptional adjustment of DNA, growth and development, signaling processes, and the coordination of multiple metabolic systems (Fukao, 2012). According to protein–protein interactions, *TIFY* members in maize can interact with genes involved in DNA replication and the cellular response to DNA damage, whereas *TIFY* family members in tomato showed significant relationships with TF MYCs and salt-responsive proteins (Figure 7). The activities of the JAZ proteins are associated with jasmonate responses, which suppress the jasmonate signals by interacting with the TFs including MYC2 and MYC3, which control the expression of downstream genes (Yan et al., 2007; Melotto et al., 2008; Bai et al., 2011). According to the results of protein–protein interactions and promoter analysis, we think that *TIFY* genes play critical roles in cell reproduction, plant growth, and dealing with stress conditions.

## Conclusion

In the current study, the *TIFY* gene family was compared between tomato (as a dicot) and maize (as a monocot) based on sequence, structure, evolutionary, expression, interaction network, and *cis*-regulatory elements. We identified 48 and 26 nonredundant putative *TIFY* genes in the genome of maize and tomato, respectively. The identified TIFYs were classified into four subfamilies (JAZ, TIFY-S, PPD, and ZML); PPD subfamily proteins were only detected in dicots. Our results revealed that all of the duplicated pairs in the *TIFY* gene family of tomato have been influenced by intense purifying selection. The amino acid residues present in the pocket sites of TIFY proteins partially differ in each subfamily, indicating that these proteins have different activities based on their ligand-binding sites. Based on the expression profile of *TIFY* genes, we found that JAZ subfamily proteins are more involved in the response to stress than other subfamilies. Key *cis*-regulatory elements were observed in the promoter site of *TIFY* genes, indicating that the *TIFY* gene family, a group of plant-specific TFs, is induced by various stimulus. Our findings demonstrate that the *TIFY* gene family plays important roles in regulating growth and development processes and inducing cell signaling in response to stress. Therefore, the results of this study can be used in future research related to the functional genomics of *TIFY* genes.

## Data Availability Statement

Publicly available datasets were analyzed in this study. This data can be found here: http://plants.ensembl.org.

## Author Contributions

PH and SF: conceptualization. SF: methodology. PH, SF, and MA: software and writing—original draft preparation. PH, MA, SA, and FM-P: writing—review and editing. FM-P: funding acquisition. All authors have read and agreed to the published version of the manuscript.

## Conflict of Interest

The authors declare that the research was conducted in the absence of any commercial or financial relationships that could be construed as a potential conflict of interest.

## Footnotes

1 http://smart.embl-heidelberg.de/webcite

2 http://pfam.sanger.ac.uk

3 https://phytozome.jgi.doe.gov/pz/portal.html

4 https://web.expasy.org/protparam/

5 https://www.genome.jp/tools-bin/clustalw

6 http://meme-suite.org/tools/meme

7 http://bioinformatics.psb.ugent.be/webtools/plantcare/html/

8 http://stringdb.org

9 http://ted.bti.cornell.edu/cgi-bin/TFGD/digital/home.cgi
