# Supplementary figures and images for: New Insights Into Structure and Function of TIFY Genes in Zea mays and Solanum lycopersicum: A Genome-Wide Comprehensive Analysis
Source: Front Genet. 2021 May 12;12:657970. doi: 10.3389/fgene.2021.657970 (PMC8155530; doi:10.3389/fgene.2021.657970)

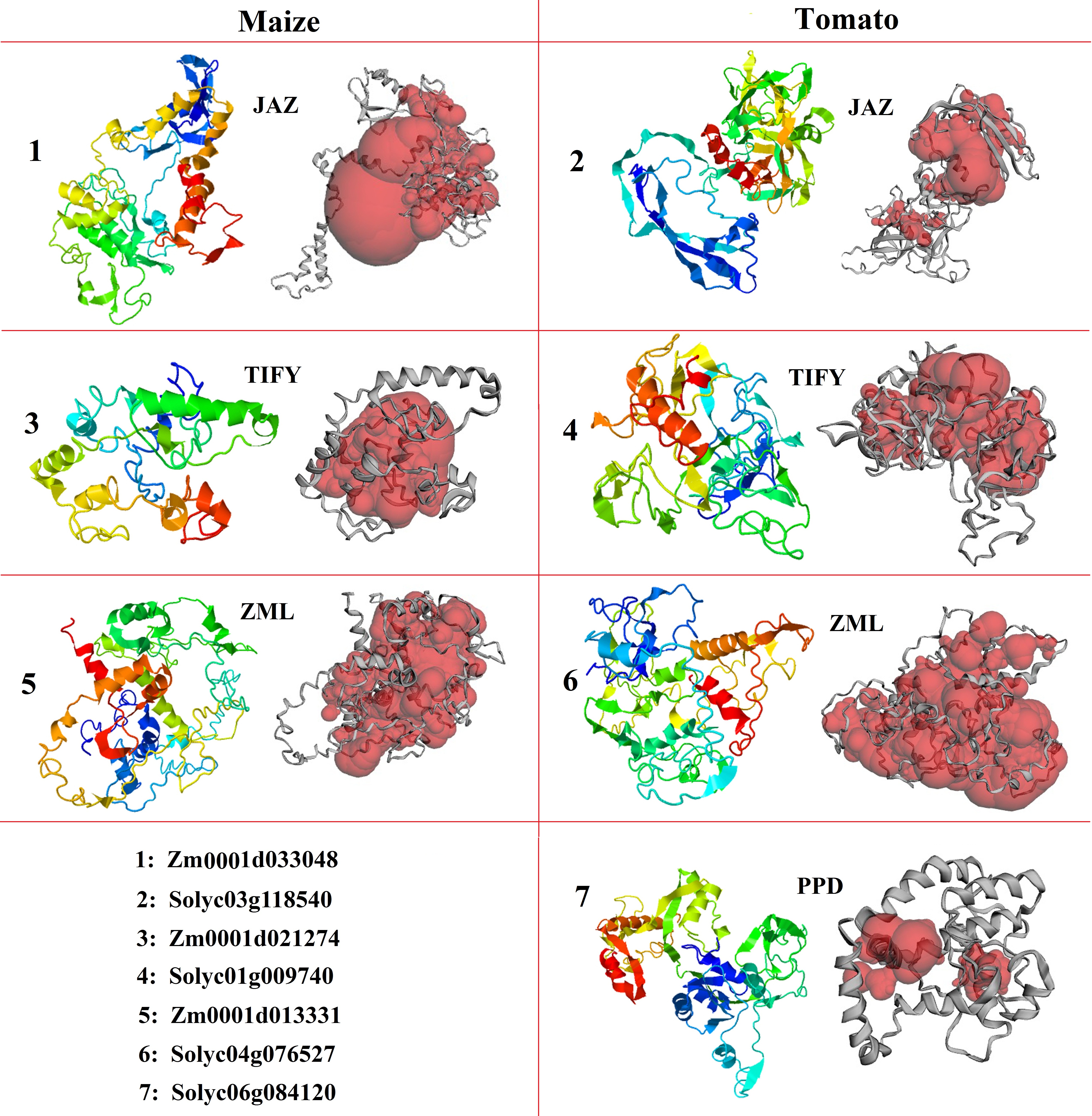

Supplement: Supplementary file 1 [file Image_1.JPEG]
